# Patterns of hospital admission with epistaxis for 26,725 patients over an 18-year period in Wales, UK

**DOI:** 10.1308/003588412X13373405386691

**Published:** 2012-05

**Authors:** SJC Fishpool, A Tomkinson

**Affiliations:** Cardiff and Vale University Health Board,UK

**Keywords:** Epistaxis, Epidemiology, Incidence, Sex, Menopause

## Abstract

**INTRODUCTION:**

Epistaxis is the one of the most common otorhinolaryngology emergencies. This study examined the age and sex distribution of all patients admitted with epistaxis to National Health Service (NHS) hospitals in Wales, UK, over a period of 18 years and 9 months.

**METHODS:**

The Patient Episode Database for Wales was examined for all patient admissions with a diagnosis of epistaxis between April 1991 and December 2009. The age and sex of these patients was recorded and the proportion of the underlying population affected was calculated by comparing admission rates to the population data derived from the 1991 and 2001 national population censuses for Wales.

**RESULTS:**

A total of 26,725 patients were admitted to NHS hospitals in Wales with epistaxis over the period studied. The proportion of the population admitted with epistaxis increased from the age of 40 onwards. For all ages except patients in the 10–14 years group, a higher proportion of the male population was admitted with epistaxis than the comparable female population. This discrepancy was most pronounced between the ages of 15 and 49 years, with the female-to-male ratio of hospital admissions with epistaxis being 0.55. These ages (15 and 49 years) approximate the average age of menarche and menopause respectively in the UK.

**CONCLUSIONS:**

Women of menstrual age have fewer hospital admissions with epistaxis. This may be due to oestrogens providing protection to the nasal vasculature (as they do to other areas of the vascular tree).

Epistaxis is the most frequently seen emergency in otolaryngology.^1^ The nature of the UK’s healthcare system affords an excellent opportunity to assess the demographics of acute epistaxis as all episodes judged severe enough by the examining physician to warrant emergency admission are admitted to a National Health Service (NHS) hospital. Furthermore, epistaxis is a relatively straightforward diagnosis to ‘code’. In this study the demographics of epistaxis in Wales were analysed over a period of 18 years and 9 months (1 April 1991 – 31 December 2009).

## Methods

The Patient Episode Database for Wales (PEDW) contains demographic and coded clinical (ICD-10)^2^ and surgical data (OPCS-4)^3^ on the care received by all patients in NHS hospitals in Wales. The PEDW was examined from 1 April 1991 to 31 December 2009. A total of 26,725 patients with epistaxis, judged by the examining clinician to be sufficiently severe to warrant hospital admission, were identified. These admissions were analysed with respect to their date of admission, and the age and sex of the patients. Data on the underlying population, taken from the 1991 and 2001 population censuses, were used as a comparison to achieve a representative estimation of admission patterns.

## Results

### Age distribution

**Figure 1:**
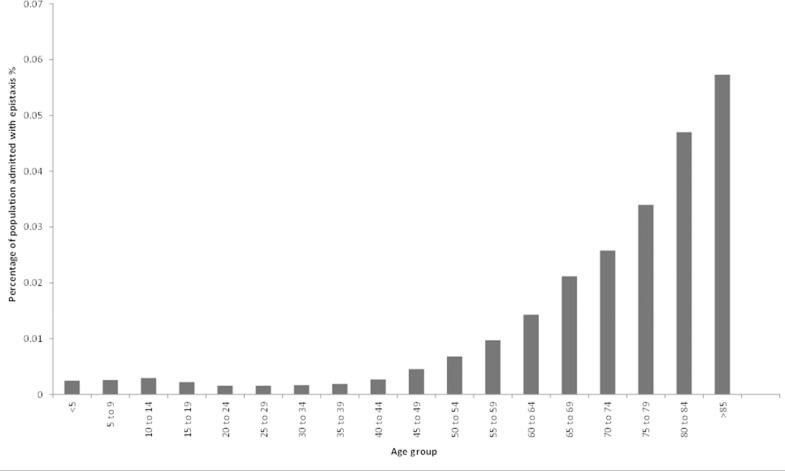
The proportion of the Welsh population admitted with epistaxis (1 April 1991 – 31 December 2009)

Figure 1 shows the epistaxis admission rate as a proportion of the underlying population number. This number is achieved by dividing the number of cases of epistaxis admitted to hospital per year in each age band by the total population of that age band. Apart from a small paediatric peak, again between 10 and 14 years of age, the incidence of epistaxis remained relatively stable until the age of 40 years, after which it increased rapidly and consistently until >85 years of age (Fig 1).

### Sex

**Figure 2:**
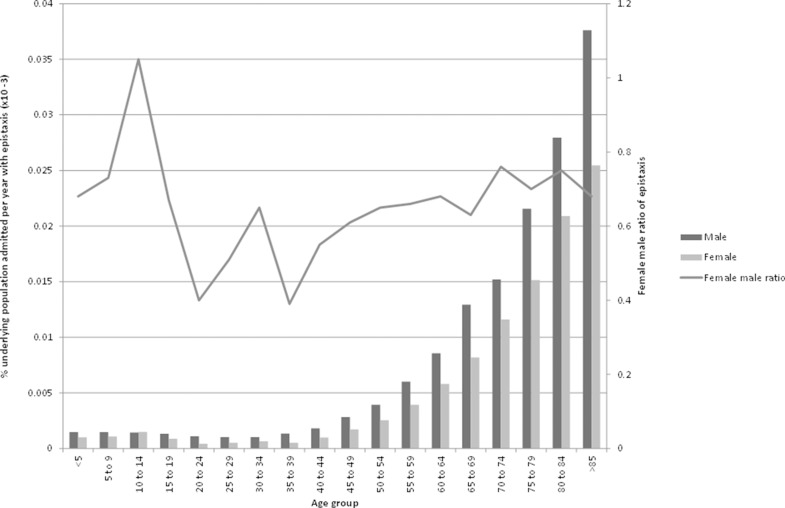
The proportion of the Welsh population admitted with epistaxis by sex (1 April 1991 – 31 December 2009)

Figure 2 demonstrates that within each five-year age group, as a proportion of their respective sex populations, males typically have more hospital admissions per year with epistaxis than females. The 10–14 years age range is the only exception to this. If the adult population is divided into a 15–49 years and a 50+ years age group (so as to roughly reflect female reproductive status), a clear pattern emerges. Approximately twice as many males aged 15–49 years are admitted to hospital with an episode of epistaxis than females of equivalent age. With the over-50s, approximately the same number of each sex were admitted to hospital with an episode of epistaxis over the same period.

### Admission rates per year

**Figure 3:**
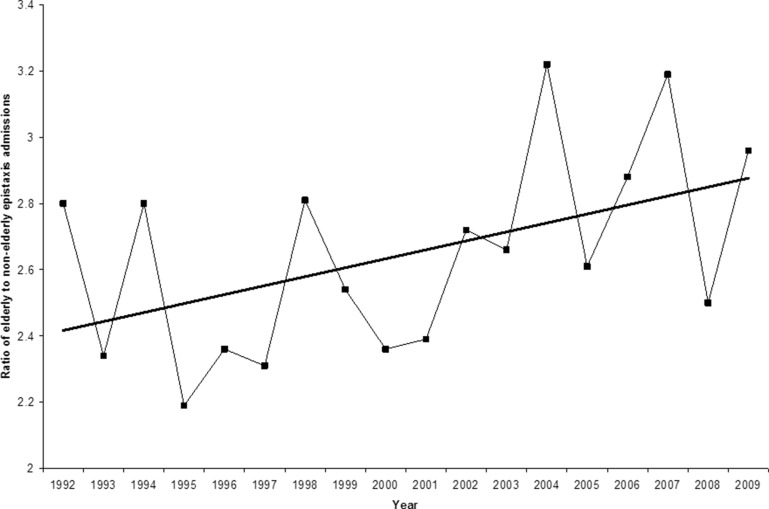
The ratio of elderly (60+ years) to non-elderly patients admitted each year with epistaxis, incorporating a linear trend line

Excluding 1991 (for which data are only available from April onwards), the number of patients admitted in Wales with acute epistaxis per year between 1992 and 2009 varied from 1,148 to 1,621 (mean: 1,440 per year). Figure 3 displays the ratio of the admissions of the elderly (defined by the World Health Organization as aged 60+)^4^ to the non-elderly.

### Duration of admission

The mean length of inpatient stay with a primary diagnosis of acute epistaxis varied from 2.8 to 3.8 days over the period 1 April 1995 to 1 April 2009 (mean: 3.2 days). The dates vary slightly from the previously examined data as the primary source of this information (PEDW) could not guarantee the reliability of length of stay data prior to 1995. It was therefore excluded from analysis. The length of stay data are recorded across a financial year by PEDW.

## Discussion

### Age distribution

The proportion of the population admitted with epistaxis rose progressively from the age range of 30–34 years onwards (Fig 1). The reasons for the increase in epistaxis admission rates with increasing age are multifactorial. They are thought to include: progressive degeneration of the tunica media in the nasal vessels with increasing age;^5^ atherosclerosis affecting nasal vessels;^6^ increasing problems with the haemostatic mechanism, regardless of concomitant drug therapy, with increasing age;^7^ a rise in anticoagulant and antiplatelet prescribing with increasing age; and a greater likelihood for the elderly to be admitted to hospital due to social circumstances regardless of the severity of the presenting condition. There is also an apparent small paediatric peak within the 10–14 age group. The reason for this is not clear from our data but possible explanations include increased digital, playground or sporting trauma.

### Sex

Males are more likely to be admitted to hospital with epistaxis than females at every age except between the ages of 10–14 years (Fig 2). This finding, derived from our series of 26,725 patients, validates the findings from an earlier study of 6,885 patients.^1^ Interestingly, this pattern of a higher incidence of haemorrhage in males is also noted in post-tonsillectomy haemorrhage.^8^

Furthermore, females of menstrual age (in the UK the average age of menarche is 13 years^9^ and the average age of the menopause is 52 years)^10^ are half as likely to be admitted to hospital with epistaxis as males of equivalent age (Fig 2). Menarche seems to be related to a fall in female epistaxis admission rates between the 10–14 and 15–19 age groups. Subsequently, the proportion of the female population admitted with epistaxis does not reach the level of the 10–14 age group again until the 45–49 age group and then, following menopause, it increases steadily in parallel with males (Fig 2).

The reasons for this relationship observed between female menstrual status and epistaxis admission rates are unknown. We propose that the female sex hormones, oestrogen in particular, have a protective effect with respect to epistaxis. In order to establish a causal relationship, the theory has to fulfil the Bradford Hill criteria,^11^ namely: plausibility, temporality, specificity, analogy, strength, biological gradient, consistency, experiment and coherence.

Certainly, the protective effect of oestrogen on epistaxis is biologically plausible. Atherosclerosis has been identified as an aetiological factor in epistaxis^5,6^ and it has been proposed that the hormonal state of the menstruating female, while protecting against cardiovascular disease in general,^12,13^ can also protect the vasculature of the nasal mucosa. Further evidence of the effect of female sex hormones on the nasal mucosa is demonstrated by the reported use of topical oestrogen for recurrent epistaxis in patients with bleeding disorders.^14^ The possibility is also raised that the use of oestrogen containing oral contraceptives in this age group may further promote a reduction in epistaxis rates, possibly due to their prothrombotic effects.^15^

Our data also suggest a temporal relationship between menarche, menopause and epistaxis admission rates in females. To ensure the specificity of the relationship, we must exclude alternative explanations for the observed relationship. Alternative explanations can be classified as behavioural and physiological. With respect to behavioural explanations, it is possible that male rates are higher due to increased traumatic epistaxis. However, while this is possible with young males, we feel it is unlikely that traumatic epistaxis makes up for the sex difference observed in middle aged patients. Perhaps females have a more stoic attitude to epistaxis than their male, age matched equivalents? However, with at least two doctors usually assessing patients before they are admitted with a diagnosis of epistaxis (an emergency physician and an ear, nose and throat doctor), we feel this explanation is unlikely. Physiological explanations may include higher blood pressure in males and a possible antithrombotic effect of male sex hormones. However, there is no evidence to support either of these arguments.

With respect to reasoning by analogy, it has already been noted that there is an increase in male post-tonsillectomy haemorrhage rates after the age of 12 years^8^ and so, from within our own specialty, we have a similar pattern from a different anatomical subsite. However, using the Bradford Hill criteria,^11^ there is obviously much more work to be done to establish a causal relationship, especially with respect to dose-response relationship, consistency, experiment, strength and coherence.

### Admission rates per year

The majority of epistaxis admissions each year are in the elderly. As the proportion of the population aged over 60 increases, there is also a gradual increase in the ratio of admissions of over-60s to under-60s (Fig 3). The overall admission rates per year vary from a low of 1,148 in 1992 to a high of 1,621 in 1998. Since 1998 there has been a downward trend in overall epistaxis admissions to 1,353 in 2009. Consequently, there seems to be an overall impression of a gentle ‘peak’ in epistaxis admission rates around 1998. Coincidentally, this coincides with the approval for usage of clopidogrel in the UK. First marketed as Plavix® by Sanofi-Aventis (Guildford, Surrey, UK) in 1998, this glycoprotein IIb/IIIa inhibitor prevents binding of fibrinogen to receptors on platelets.

However, prescribing data obtained from NHS Wales Prescribing Services, unfortunately only available from 2001 onwards, show clopidogrel prescriptions peaking in 2005. Furthermore, prescribing rates were significantly lower in the earlier years of our available data. It therefore seems unlikely that the introduction of clopidogrel has had a major effect on epistaxis rates.

### Duration of admission

The mean duration of an admission with epistaxis has remained fairly constant from 1995 to 2009 around a mean of 3.2 days. This is in spite of the introduction of a number of new methods, both indirect and direct, for the management of epistaxis in the study period.

In terms of indirect methods of epistaxis control, the Rapid Rhino® nasal pack (ArthroCare, Austin, TX, US) was introduced in 2000. It is now used widely in the management of epistaxis across Wales and the UK. The manufacturer claims greater comfort of insertion and removal of this nasal pack, and this is supported by some studies^16,17^ but it would appear that it has not shortened the average inpatient stay. With respect to direct methods of epistaxis arrest, the popularisation of endoscopic surgical techniques, most notably endoscopic sphenopalatine artery ligation first described in 1992,^18^ does not seem to have reduced inpatient stay either. Although undoubtedly useful for fortunately rarer cases of refractory epistaxis, it would appear this technique does not appear to have had an impact on the duration of admission for the majority of patients.

## Conclusions

This study suggests that rates of hospital admission for epistaxis increase with increasing age. The reasons for this are multifactorial. Furthermore, women of menstrual age seem relatively protected from epistaxis. One explanation may be that female sex hormones (both physiological and pharmacological) have a protective effect on the nasal vasculature. With respect to overall admission rates with a primary diagnosis of epistaxis, these are falling slowly from a peak in 1998. However, the mean duration of inpatient stay has remained stable despite the introduction of new management techniques.
